# Reference values of amino acids, acylcarnitines and succinylacetone by tandem mass spectrometry for use in newborn screening in southwest Colombia

**DOI:** 10.25100/cm.v48i3.2180

**Published:** 2017-09-30

**Authors:** Nora Céspedes, Angela Valencia, Carlos Alberto Echeverry, Maria Isabel Arce-Plata, Cristóbal Colón, Daisy E Castiñeiras, Paula Margarita Hurtado, Jose Angel Cocho, Sócrates Herrera, Myriam Arévalo-Herrera

**Affiliations:** 1 Malaria Vaccine and Drug Development Center (MVDC), Cali, Colombia; 2 Laboratorio de Metabolopatías, Hospital Clínico Universitario de Santiago, Santiago de Compostela, España.; 3 Facultad de Ciencias de la Salud, Pontificia Universidad Javeriana, Cali, Colombia; 4 Centro Médico Imbanaco, Cali, Colombia; 5 Asoclinic Inmunología Ltda, Cali, Colombia; 6 Facultad de Salud, Universidad del Valle, Cali, Colombia

**Keywords:** Neonatal Screenings, inborn errors, amino acids, acylcarnitines, Succinylacetone, Mass Spectrometry, Tandem, Tamizaje neonatal, errores innatos, aminoácidos, acilcarnitinas, succinilacetona, espectrometría de masas en tándem

## Abstract

**Introduction:**

Inborn errors of metabolism (IEM) represent an important public health problem due to current diagnosis and treatment limitations, poor life quality of affected patients, and consequent untimely child death. In contrast to classical methods, tandem mass spectrometry (MS/MS) has allowed simultaneous evaluation of multiple metabolites associated with IEM offering higher sensitivity, low false positive rates and high throughput.

**Aims:**

Determine concentration levels for amino acids and acylcarnitines in blood of newborns from Colombia, to establish reference values for further use in diagnosis of IEM.

**Methods:**

Implementation of a method to determine amino acids, acylcarnitines and succinylacetone in newborn dried blood spots using MS/MS, and its application in a cross-sectional study conducted in 891 healthy neonates from Cali and Quibdo cities is described.

**Results:**

fifty-seven analytes that allow the diagnosis of more than 40 different pathologies were tested. The method showed to be linear, precise and accurate. Healthy neonates 1-18 days of age were included, 523 from Cali and 368 from Quibdo; 52% male and 48% female. Age-related differences on the concentration levels of amino acids and acylcarnitines were observed whereas no significant differences by gender were found.

**Conclusion:**

The study has contributed to reveal the usual concentration levels of amino acids, acylcarnitines and succinylacetone that could be used as reference for the establishment of a newborn metabolic screening program in Colombia.

## Resumen

**Introducción:**

Los Errores Innatos del metabolismo (EIM) representan un importante problema de salud pública debido a limitaciones en el tratamiento y diagnóstico oportuno, la pobre calidad de vida de los pacientes afectados, así como la muerte infantil prematura. Comparada con los métodos clásicos, la espectrometría de masas en tándem (MS/MS) ha permitido la evaluación simultánea de múltiples metabolitos asociados con EIM, con una alta sensibilidad, baja proporción de falsos positivos y alto rendimiento.

**Objetivos:**

Determinar los niveles de concentración de aminoácidos y acilcarnitinas en sangre de recién nacidos de Colombia, para establecer los valores normales para usarlos como referencia en el diagnóstico de EIM.

**Métodos:**

Aquí, se describe la implementación de un método para determinar aminoácidos, acilcarnitinas y succinilacetona en gotas de sangre seca de recién nacidos usando MS/MS, y su aplicación en un estudio de corte transversal realizado en 891 neonatos sanos de las ciudades de Cali y Quibdó.

**Resultados:**

Se evaluaron 57 analitos que permiten el diagnóstico de más de 40 patologías diferentes. El método mostró ser lineal, preciso y exacto. Se incluyeron neonatos sanos de 1-18 días de edad, 523 de Cali y 368 de Quibdó, 52% hombres y 48% mujeres. Se observaron diferencias en los niveles de concentración de aminoácidos y acilcarnitinas relacionadas con la edad, mientras que no se encontraron diferencias significativas por sexo.

**Conclusión:**

El estudio ha contribuido a revelar los niveles usuales de concentración de aminoácidos, acilcarnitinas y succinilacetona que pueden ser usados como referencia para el establecimiento del programa de tamizaje neonatal metabólico en Colombia.

## Introduction 

The Inborn Errors of Metabolism (IEM) are part of the group of so-called "rare, forgotten or orphan diseases" that fortunately affect very few people in the world. The incidence could be estimated in one person per 2,000 inhabitants for the most common, but in some cases can be as low as one per 10,000 or more inhabitants, which makes these diseases to be poorly known, seldom studied and treated and therefore neglected ^1^ ^,^ ^2^. It is estimated that IEM could affect 6-8% of the world population, however, the data vary from country to country and in some like Colombia no data are available due to lack of epidemiological studies and the restrictions for accurate diagnosis. 

IEM are serious, degenerative, chronic diseases with painful and disabling clinical manifestations ranging from unnoticed clinical pictures or confused with other diseases to varying degrees of mental retardation and physical disability ^3^. These diseases can result in death at an early age, creating a considerable family and social burden. The greatest problem with these diseases is the delay in diagnosis (5-10 years) or misdiagnosis due to the lack of specialized laboratories that perform accurate tests, leading to a delayed or lack of treatment. When available, early diagnosis and adequate treatment allow patients to lead an almost normal life, reducing sequelae or at least substantially lessening organ damages.

Tandem mass spectrometry (MS/MS) is a technology that allows simultaneous detection and identification of multiple analytes with high sensitivity, accuracy and precision, with high specificity. Based on these properties, MS/MS has been incorporated as a diagnosis tool for IEM screening ^4^ in a screening system capable of detecting over 50 different conditions ^5^. First demonstrated by Millington *et al*. ^6^ ^,^ ^7^, and further optimized and applied to metabolic neonatal screening by different groups ^8^ ^,^ ^9^ including de addition of succinylacetone (SUAC), as a specific marker for tyrosinemia type I (Tyr I) ^10^ ^,^ ^11^, MS/MS currently replaces traditional screening techniques that usually analyse individual biomarkers for each disease. At present, MS/MS is routinely used in developed countries as United States, Canada, Germany and Spain ^12^ ^-^ ^15^, and in Latin America countries such as México, Brazil and Costa Rica ^16^ ^-^ ^18^. In Colombia there is not enough awareness about this issue, which leads to a lack of clarity regarding public health policies for their management ^19^. Currently, only congenital hypothyroidism is included in the list of mandatory diseases for screening, however it is known that screening is not performed in most regions of the country. 

Because of the urgent need for a highly sensitive method for diagnostic and efficient IEM screening in Colombia, this study is focused on the establishment of concentration values of amino acids (AAs), acylcarnitines (ACs) and succinylacetone (SUAC) in a sample of Colombian newborns using MS/MS technology.

## Materials and Methods

### Subjects

A total of 891 healthy newborns from Cali (n= 523, department of Valle del Cauca) and Quibdo (n= 368, department of Choco), were recruited between August 2012 and July 2015 and included in the study. Blood specimens were collected by heel-stick and spotted on filter paper cards (Whatman nº903 filter, GE Healthcare, Westborough, USA) which were dried for 24 hours at room temperature and then stored at 4° C until use, according to the standards from Clinical and Laboratory Standard Institute ^20^. Duplicate samples were stored frozen at -20° C with a drying agent. This trial was conducted according to ICH E-6 Guidelines for Good Clinical Practices and the protocol was approved by Institutional Review Boards (IRB) of the Malaria Vaccine and Drug Development Center-MVDC (CECIV from Cali, act number 005), Clínica Versalles (Cali) and Hospital San Francisco de Asís (Quibdo). Written informed consent (IC) was obtained from the mother of each newborn at enrollment.

### Inclusion criteria

All infants included in the present study fulfilled all selected inclusion criteria to ensure that they were not suffering from any disorder or disease. Among those criteria, we highlighted that healthy male and female newborns had to have weights in the range of 2,500 -4,000 g, gestational ages of 37-42 weeks, an APGAR score greater than 7 at 10 min and aged between 2 and 18 days.

### Materials

Isotopically labeled internal standards AAs and ACs were purchased from Cambridge Isotope Laboratories, Inc.; hydrazine sulfate salt and HPLC grade acetonitrile from Sigma-Aldrich, SUAC from HT Brink, University hospital of Amsterdam, formic acid and 3 N HCl in *n*-butanol from Fluka. The filter paper 903 Protein Saver used for blood spot samples were purchased from Whatman. Base, low, medium and high level blood spot controls were supplied by the Newborn Screening Quality Assurance Program of Center of Disease Control and Prevention (CDC) (Lot:1421-1424; 1462-1464). Dried blood samples were analyzed using high performance liquid chromatography (Shimadzu Scientific Instruments, Columbia, MD) coupled to Tandem mass spectrometer 3200 QTRAP (AB Sciex). 

### Sample preparation

Dried blood spots (DBS) were punch at 3.2 mm diameter and placed into a single well of a polystyrene 96-well plate, to which 120 μL were added of a daily working solution containing acetonitrile: water (80:20 v/v), 0.08% formic acid, 3.6 mol/L hydrazine hydrate and internal standards of AAs, ACs and SUAC. The plate was sealed using adhesive Sealing Film (Fisherbrand No 08-408-240) and shaken at 600 rpm for 45 min at 45º C. The extracts were transferred to a new polystyrene 96-microwell plate and then dried under N_2_ atmosphere at room temperature for approximately 25 min at 40° C. Then 50 µL of methanol were added and dried again for 10 min. Samples were then reconstituted in 60 μL of n-butanol 3N HCl and incubated at 65º C ± 5° C for 20 min. The resulting mixtures were again dried for about 20 min, and each residue was finally reconstituted in 150 μL of mobile phase (acetonitrile: water 50:50 containing 0.025% formic acid), covered with aluminum foil, shaken for 10 min at room temperature, centrifuged at 500 r.p.m. for 4 min and placed into an autosampler tray for MS/MS analysis.

### MS/MS analysis

A triple quadrupole tandem mass spectrometer, operated in positive-ion mode was used for analysis of AAs, ACs and SUAC. The samples were run using isocratic mobile phase (acetonitrile: water - 50:50 containing 0.05% formic acid) with a flow rate of 70 μL/min in 3 min run per sample using a high-performance liquid chromatography pump. The analysis was done using three different experiments per run: 1. Neutral loss of m/z 102 (scan range m/z: 130-280) for AAs detection, 2. Precursor ion of m/z 85 (scan range m/z: 200-550) for ACs and the multiple reaction mode (MRM) for SUAC (m/z 211 → 137) and the AA with lower sensitivity such as Arg (m/z 231 → 70), Gly (m/z 132 → 76), Leu (m/z 188 → 86), Met (m/z 206 → 104), Ser (m/z 162 → 60), His (m/z 156 → 110), OH-Pro (m/z 188 → 68), Thr (m/z 176 → 74) Ornitine (m/z 189 → 70) and Citruline (m/z 232 → 113). The software Analyst 1.5.2 (BioSciex) was used for data acquisition and Chemoview and Neoscreen were used for data analysis. Quantification of target analytes was achieved by calculating the ion abundance ratios of each pure compound relative to isotopically labeled internal standards (IS).

### Linearity and limit of detection 

Linearity of the method for AAs, ACs and SUAC was estimated in duplicated by inter-assay analysis of the DBS calibrators from CDC at four different concentrations between 3.7 and 746.7 µmol/L for AAs, 0.1 and 52.8 µmol/L for ACs and 0.3 and 11.1 µmol/L for SUAC and at 95% of confidence interval (CI). The limit of detection (LOD) was evaluated using a linearization in three different lower levels of concentration for each analyte, taking into account the baseline noise as an indicator of instrument sensitivity. 

### Precision and accuracy

Both the intra-assay and inter-assay precision for AAs, ACs and SUAC was estimated at two medium concentration levels of the DBS calibrators from CDC. Intra-assay precision was determined by duplicates in five repetitions, whereas the inter-assay precision was determined in duplicates for three days and two different analysts. Precision was denoted as the percent of coefficient of variation (%CV <20%) and Cochran values (<0.555) for intra-assay and inter-assay precision, respectively. 

The accuracy of the assay was determined using DBS calibrators from CDC at four concentration levels (Lot: 1321-24 for AA and Lot: 1361-64 for AC) over three batch runs. Accuracy was denoted as the relative error (%RE). Accuracy was required to be within ±20%RE ^21^.

### Statistical analysis

Data were processed with R-project by Bell Laboratories (formerly AT&T, now Lucent Technologies). Results are expressed as coefficient correlation (R^2)^ for linearity, critical value of the F-distribution (ANOVA analysis) for analysis of variance, CV and Cochran values for precision and percentage of recovery for the Accuracy. A Shapiro-Wilk Test was used to check normality; however, there was not enough evidence to say that the data was normally distributed. Group comparisons by gender and study site were determined by a Mann Whitney U test and correlations between age and each analyte concentration were tested with a Spearman test. A *p* value ≤0.05 was considered significant.

## Results

### Validation of the method

Linearity was determined using four levels of concentrations. Coefficients of linear regressions (R²) for AAs, ACs and SUAC were >0.96 for all analytes (Table 1). Linearity ranges were within the ranges established by the CDC. The analysis of variance for the regression of each analyte by ANOVA test showed critical values of the F-distribution much lower than 5% for all analytes (Table 1). The mean intra-assay precision over the entire concentration range was always less than 20%, and the inter-assay precision, measured as the Cochran value (0.555) was compiled for all analytes (Table 1).

**Table 1:** Validation of the method

Analyte	LOD* (µmol/L)	Linear range (µmol/L)	Linearity	ANOVA	Precision		Accuracy
			**^_R2_^	†*Critical values F*	¶CV	Cochran	§% RE
			≥0.95	<5%	≤ 20%	0.555	80 - 120%
Phe	0.10	71.8-325.4	0.998	3.399E-05	8.17	0.430	83.24
XLeu	1.43	116.0-445.7	0.966	2.681E-03	10.39	0.245	91.06
Met	4.97	11.4-214.2	0.980	1.236E-03	14.65	0.309	96.59
Tyr	0.33	49.7-489.4	0.993	2.567E-04	6.18	0.356	98.63
Val	2.27	121.4-428.6	0.991	3.389E-04	6.70	0.534	92.47
Cit	0.35	26.3-232.7	0.999	3.050E-07	4.78	0.455	109.94
Ala	1.50	292.8-625.5	0.978	1.374E-03	6.17	0.250	105.20
Arg	0.89	10.0-266.1	0.995	1.358E-04	5.90	0.484	84.34
SUAC	0.12	1.4-9.0	0.995	1.318E-04	6.78	0.374	104.79
Free carnitine (C0)	0.04	25.9-79.0	0.971	2.104E-03	9.28	0.277	92.58
Acetyl (C2)	0.08	12.9-39.4	0.939	6.500E-03	10.95	0.220	83.78
Propionyl (C3)	0.14	1.3-12.4	0.999	8.953E-06	8.63	0.384	114.48
Butiryl (C4)	0.09	0.2-4.4	0.992	3.003E-04	13.19	0.297	94.55
Isovaleryl (C5)	0.01	0.1-2.4	0.999	7.922E-06	5.07	0.544	105.48
Glutaryl (C5DC)	0.15	0.5-1.8	0.997	6.015E-05	13.64	0.336	88.96
3-OH-valeryl (C5OH)	0.01	0.5-3.4	0.964	2.975E-03	5.05	0.304	79.77
Hexanoyl (C6)	0.01	0.0-2.2	0.999	2.258E-06	8.82	0.554	113.21
Octanoyl (C8)	0.06	0.0-2.3	0.983	9.102E-04	2.80	0.353	116.59
Decanoyl (C10)	0.03	0.0-2.5	0.981	1.165E-03	7.37	0.332	113.11
Dodecanoyl (C12)	0.01	0.0-2.1	0.991	3.672E-04	12.37	0.366	100.13
Myristoyl (C14)	0.01	0.1-2.6	0.997	6.014E-05	12.66	0.467	107.10
Palmitoyl (C16)	3.47*	0.9-9.2	0.958	3.752E-03	8.14	0.330	113.30
Hydroxy-Palmitoyl (C16OH)	2.83*	0.0-0.9	0.995	1.664E-04	8.17	0.43	175.40
Stearoyl (C18)	4.58*	0.6-5.7	0.996	9.952E-05	10.39	0.245	91.06
OH-stearoyl (C18OH)	3.75*	0.0-1.4	0.987	6.520E-04	14.65	0.309	96.59

* LOD: Límite de detección

**R2: Correlation Coeficient

† F: Citic value of distribution F

¶ CV: Variation Coeficient

§ %RE: Relative Error

Accuracy of the method was determined by replicated analysis of samples containing known amounts of each analyte in DBS calibrators from CDC. The accuracy data obtained were in the range of 83.2 to 109.9% for AAs, of 80.0 to 116.6% for ACs and was 104.8% for SUAC (Table 1). The LODs obtained for AAs, ACs and SUAC are also summarized in Table 1, showing the lowest detection for phenylalanine (Phe) of 0.1 µmol/L and 3-hidroxypalmitoylcarnitine (C16OH) of 2.83 nmol/L.

### Concentrations of AAs, ACs and SUAC in the studied population

A total of 891 healthy neonates between 1 and 18 days of age, 463 (52%) male and 428 (48%) female, were included in this study. Of them, 313 neonates were between 1-2 days old, 257 between 3-8 days old and 321 were 9 to 18 days old.

A total of 25 analytes were used in the validation process, which form part of quality controls given by the CDC. In addition, 32 other analytes were tested to complete a total of 57 analytes of which 19 were AAs, 37 were ACs and SUAC, thus allowing diagnosis of 50 different pathologies. Specific markers were selected according to information obtained from Regions 4 Genetic New Born Screening ^22^ and transferred from Hospital Clínico Universitario, Santiago de Compostela, España to Malaria Vaccine and Drug development Center in Cali (Colombia).

In general, the AAs (n= 8, Table 2) had mean concentrations ranging between 15 and 247 µmol/L. Of them, xLeu (mean 247 µmol/L), Ala (192 µmol/L) and Val (128 µmol/L) were the most abundant AAs, whereas Citruline (15 µmol/L) and Arg (20 µmol/L) were the less abundant. In general, ACs with shorter chains were the most concentrated whereas those of longer chain were the less abundant. Higher concentrations were observed for free carnitine (32 µmol/L) and acetyl carnitine (23 µmol/L), whereas hydroxystearoyl carnitine (C18-OH) (0.07 µmol/L) were the less concentrated. Palmitoyl carnitine (C16) was the most abundant among those of long-chains (Table 2). 

**Table 2:** Reference concentration values for AAs and ACs.

Analyte	MEAN (µmol/L)	SD	P_1_*	P_99_**
Phe	44.27	11.78	23.92	78.94
XLeu	246.72	121.16	72.61	536.72
Met	35.50	19.71	11.00	101.91
Tyr	96.27	34.72	37.03	201.25
Val	128.42	32.10	59.18	204.00
Cit	14.95	4.56	7.81	27.52
Ala	192.01	53.86	91.43	348.82
Arg	19.94	11.48	4.40	52.07
C0	32.39	13.71	13.63	78.74
C2	22.69	8.62	9.02	54.31
C3	1.24	0.73	0.23	4.05
C4	0.22	0.15	0.07	0.64
C5	0.24	0.09	0.09	0.59
C5DC	0.12	0.07	0.03	0.36
C5OH	0.22	0.09	0.10	0.55
C6	0.16	0.09	0.04	0.51
C8	0.16	0.08	0.05	0.43
C10	0.17	0.09	0.05	0.45
C12	0.27	0.16	0.08	0.81
C14	0.32	0.15	0.10	0.84
C16	2.48	1.19	0.72	6.17
C16OH	0.10	0.06	0.02	0.27
C18	0.83	0.36	0.30	2.00
C18OH	0.07	0.05	0.02	0.26

SD: standard deviation

*Percentile 1 lower acceptance limit

**Percentile 99 upper acceptance limit

### Age and gender distribution

Normal concentrations of AAs and ACs were compared with regard to factors such as age and gender. All data sets did not show a normal distribution when they were evaluated by Shapiro-Wilk test for normality, therefore non-parametric tests were used to check for differences between concentrations of AAs and ACs regarding different factors.

**Figure 1:**
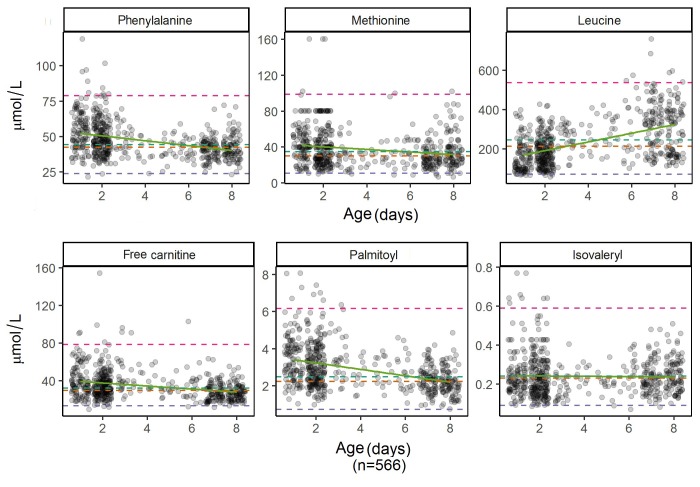
Scatter plots for some A. amino acids (phenylalanine, methionine and leucine) and B. short-chain carnitine (free carnitine and isovalerylcarnitine) and long-chain carnitine (palmitoylcarnitine) by age. Mean, median, 1th and 99 percentiles and the regression line are highlighted. Each analyte has a different scale all measured in µmol/L.

Age had a significant effect on analytes concentration, with rho ranging from 0.02 to 0.52 for positive relations and -0.06 to -0.39 for negative relations (Table 3). For the AAs, only phenylalanine and methionine had a negative correlation (Fig. 1A), whereas for the ACs, in all cases but Isovaleryl (C5, rho= 0.02), the correlation between the concentration and age was negative (rho <0) (Fig. 1B, Table 3), indicating that the concentration of these analytes decreases slightly with age, while it increases with age for isovaleryl and most of the tested AAs. 

**Table 3:** Statistical analysis according to age (Spearman's correlation) and gender (Mann Whitney U test).

Analyte	Spearman Correlation		Female		Male		Mann Whitney U Test
	rho	*p*-values	Median (µmol/L)	IQR*	Median (µmol/L)	IQR1	p-values
Phe	-0.38	9.30E-21	42.34	36.14-49.44	42.21	36.09-49.43	0.842
XLeu	0.49	5.05E-35	216.36	149.29-335.02	213.10	149.91-347.67	0.677
Met	-0.21	8.99E-07	30.42	21.25-43.53	28.83	21.5-40.04	0.633
Tyr	0.46	6.78E-31	93.84	72.67-115.06	92.17	71.08-115.74	0.467
Val	0.50	2.10E-37	127.77	106.77-150.94	128.84	105.28-147.00	0.535
Cit	0.05	2.03E-01	14.06	12.02-17.00	14.30	12.12-16.89	0.955
Ala	0.30	1.69E-13	190.78	155.77-229.23	183.80	155-217.67	0.139
Arg	0.52	2.24E-40	16.94	9.73-26.00	18.83	10.83-29.60	0.018
C0	-0.29	1.45E-12	28.34	22.24-36.87	30.92	24.46-38.66	0.004
C2	-0.28	1.90E-11	21.03	17.30-26.59	20.95	17.18-25.61	0.484
C3	-0.12	4.96E-03	1.04	0.78-1.44	1.10	0.82-1.48	0.133
C4	-0.10	1.81E-02	0.20	0.15-0.27	0.20	0.15-0.26	0.798
C5	0.02	6.19E-01	0.23	0.17-0.28	0.23	0.18-0.28	0.945
C5DC	-0.32	4.95E-14	0.10	0.06-0.14	0.10	0.07-0.15	0.107
C5OH	-0.10	1.29E-02	0.20	0.16-0.26	0.21	0.16-0.26	0.252
C6	-0.31	1.26E-13	0.14	0.10-0.18	0.14	0.10-0.20	0.573
C8	-0.31	2.32E-13	0.14	0.10-0.19	0.14	0.11-0.20	0.251
C10	-0.35	1.10E-17	0.15	0.10-0.20	0.15	0.11-0.21	0.086
C12	-0.38	1.57E-20	0.22	0.15-0.32	0.23	0.16-0.34	0.557
C14	-0.31	1.99E-14	0.28	0.21-0.37	0.31	0.22-0.39	0.024
C16	-0.39	2.23E-22	2.22	1.61-3.06	2.25	1.70-3.12	0.310
C16OH	-0.36	3.95E-17	0.08	0.05-0.13	0.08	0.06-0.12	0.560
C18	-0.36	3.97E-19	0.75	0.56-0.99	0.75	0.59-1.00	0.727
C18OH	-0.28	2.16E-10	0.06	0.04-0.09	0.06	0.04-0.09	0.938

*IQR is the range between percentile 25 and 75

**Figure 2:**
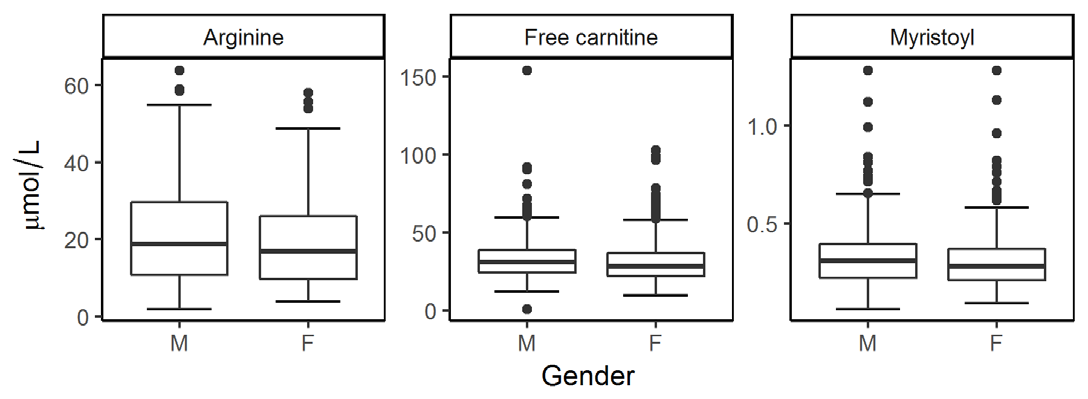
Box-whisker plots of analytes by gender for arginine, free carnitine and myristoylcarnitine. The boxes correspond to 25^th^ and 75^th^ percentile and the horizontal line is the median concentration for the analyte.

When comparisons by gender were made, arginine was the only amino acid showing significant differences (*p*= 0.018, higher concentration in males), while, in the case of the ACs, free carnitine and myristoyl carnitine also presented significant differences with higher concentrations in males, with *p*-values of 0.004 and 0.024, respectively (Fig. 2, Table 3).

## Discussion 

Herein, we reported a methodology to simultaneously evaluate 57 analytes related to AAs, ACs and SUAC and associated with more than 40 IEMs, using automated tandem mass spectrometry (MS/MS) on dried blood spots collected from Colombian newborns. This methodology revealed the normal concentrations values of these analytes in an ethnically diverse population of 891 newborns. Based on the national birth rate of the country (663,908 birth/year) ^23^, this sample set represents a statistically significant population for the establishment of normal values for the analyzed metabolites that meets the requirements of the Clinical and Laboratory Standards Institute (CLSI) guidelines ^24^. Results indicated that blood samples enriched with each analyte in filter paper spots (Quality control from CDC) and subsequently mixed with labelled internal standards (isotope dilution method) led to data linearity over a wide range of concentrations, as well as to low detection limits. 

Limitation in samples collections, such as a suitable collection time, appropriate application of heel-stick blood to filter paper, sufficient drying before packaging, and timely shipment to the screening laboratory, was present in less than 10% of the samples. However, we found that the validation parameters established for this method displayed great precision and accuracy leading to CVs and recoveries according to internationally accepted values for bioanalytical methods to be considered reliable and reproducible ^21^. The variables used in the regression for each analyte were statistically significant with critical values of the F-distribution < 5% (ANOVA test), demonstrating therefore that the model implemented here was adequate. The usual concentration values calculated from all samples showed a higher cutoff for AAs and lower for ACs and SUAC, which was in agreement with the highest intake of proteins during infant feeding. In comparison with 1^st^ and 99^th^ percentile reported in Region 4 genetics for these metabolites, we found values in similar ranges for most of them using a much smaller population. Some carnitines including and Hidroxy-octadecanoylcarnitine (C18), showed much greater 99^th^ percentile values compared with those reported by other laboratories, which could be a problem due to a possible increase of false negatives by overlapping between the higher cut-off of healthy infants and lower cutoff of sick infants. Although ACs mean concentrations were in a low range of 0.07-32.4 µmol/L, these were always above their LOD, which is important for diagnostic distinction from patients with some metabolic disorders ^25^. 

Acylcarnitine profiles of dried blood samples were characterized by a decrease in the concentration of most of them as the child aged, consistent with data reported in previous studies ^26^. In the case of isovaleryl carnitine, which showed a slight increase in concentration with age, it could be attributed to its variability depending on external factors such as the use of antibiotics or hemolysis related to jaundice. Age should be considered an important factor in determination of reference concentration values, taking into account challenges related to diagnosis in older infants due to decreasing AC concentrations. The lower cut off values for these analytes could lead to a difficulty in diagnosing metabolic disorders that show carnitine deficiency. No significant differences were found between males and females for almost all metabolites, except for arginine, free carnitine and myristoylcarnitine, which showed a higher concentration for male neonates, in agreement with results previously reported for carnitines ^27^ ^,^ ^28^. 

Significant differences were observed between levels of analytes when Cali and Quibdo results were compared (data no shown). However, there were also differences in the mean age of the newborn enrolled in the two populations, while the newborn from Cali had a mean of 9 days (range: 4-18 days) age, Quibdo's newborn had a mean age of 2 days (range: 1-7 days). It is known that the age, drugs therapies, parenteral nutrition, transfusions and type of feeds can all potentially influence the results. These differences were taking into account when cut-off values were established.

Use of MS/MS for Newborn screening of IEM offer some advantages such as analytical sensitivity, selectivity and accuracy, with the possibility to measure several analytes in a single analysis and relatively low rate of interferences ^29^. Most of the limitations of the method presented here are related to the DBS, namely, blood sampling, chromatographic and volume effects, analyte stability and hematocrit effects ^30^, that in some cases, such as the inherent interpatient variation in the volume of blood contained in the blood spot punch, can be solve by strict adherence to the general standard guidelines ^20^. It has been suggested, that the integrity of dried blood spots can be compromised within a short time frame by humidity and temperature during transportation of the samples, a significant degradation of AAs and ACs in the blood spot at high temperature (45° C) and humidity (>70%), particularly in the first day of storage ^31^. Therefore, transportation and storage condition must be controlled. Moreover, most metabolites used for newborn screening depend on hematocrit and on position of the disk ^32^ ^,^ ^33^. Although hematocrit levels in neonates are significantly variable (45-60 %), in most cases, metabolite concentrations exceed unequivocally their respective cut-off levels and hematocrit and/or location of disk do not significantly affect the diagnosis. However, it is important to clarify that within the methodology of our study, no hematocrit was measured before the metabolic tests.

In conclusion, this is the first report of analyses of characteristic metabolites for IEM diagnosis by MS/MS in Colombia such as it is done in developed countries. At present, the biochemical tests and analytical tools being used in Colombia do not have enough sensitivity and specificity when compared to MS/MS. Additionally, those techniques such as gas chromatography, and enzymatic and colorimetric methods use complex protocols with long treatment of samples and are restricted to a limited number of analytes associated with a few diseases. The application of advanced technology, such as automated electrospray MS/MS analysis, allowed detection of more than 40 IEM with a significant reduction in analysis time (2-3 min). Data reported here represents a significant contribution to establish normal concentration levels of AAs, ACs and SUAC to be used as reference for implementation of a new newborn metabolic screening program in Colombia based on MS/MS technology, allowing to define the national prevalence of IEM.
