# Supplementary material for: Reference values of amino acids, acylcarnitines and succinylacetone by tandem mass spectrometry for use in newborn screening in southwest Colombia
Source: Colomb Med (Cali). 2017 Sep 30;48(3):113–9. doi: 10.25100/cm.v48i3.2180 (PMC5687862; doi:10.25100/cm.v48i3.2180)
Supplement: Translation

# Translation: Valores de referencia de aminoácidos, acilcarnitinas y succinilacetona por espectrometría de masas en tándem para su uso en el tamizaje neonatal en el suroccidente de Colombia

## Introducción

Los errores innatos del metabolismo (EIM) son parte del grupo de las llamadas "enfermedades raras, olvidadas o huérfanas" que por fortuna afectan a muy pocas personas en el mundo. La incidencia puede estimarse en una persona por cada 2,000 habitantes para las enfermedades más comunes, pero en algunos casos puede ser tan baja como uno por cada 10,000 o más habitantes, lo que hace que estas enfermedades sean poco conocidas, estudiadas y tratadas, y como consecuencia desatendidas ^1^^,^^2^. Se estima que los EIM podrían afectar entre el 6-8% de la población mundial, sin embargo, los datos varían de un país a otro y en algunos como Colombia no hay datos disponibles debido a la falta de estudios epidemiológicos y las restricciones para un diagnóstico preciso.

Los EIM son enfermedades crónicas graves y degenerativas, con manifestaciones clínicas dolorosas e incapacitantes que van desde cuadros clínicos inadvertidos o que se confunden con otras enfermedades, con diversos grados de retraso mental y discapacidad física ^3^. Estas enfermedades pueden causar la muerte a una edad temprana, lo que crea una carga familiar y social considerable. El mayor problema con estas enfermedades es el diagnóstico tardío (5-10 años) o erróneo debido a la falta de laboratorios especializados que realizan exámenes precisos, lo que conduce a un retraso o falta de tratamiento. El diagnóstico precoz y el tratamiento adecuado y oportuno de estas enfermedades permiten a los pacientes llevar una vida casi normal, reduciendo las secuelas o al menos reduciendo sustancialmente los daños en los órganos.

La espectrometría de masas en tándem (MS/MS) es una tecnología que permite la detección e identificación simultánea de múltiples analitos con alta sensibilidad, exactitud y precisión, con alta especificidad. Basándose en estas propiedades, se ha incorporado la MS/MS como una herramienta de diagnóstico para el tamizaje de EIM ^4^ en un sistema de capaz de detectar más de 50 analitos diferentes ^5^. Millington et al ^6^^,^^7^ fue el primero en desarrollar el método de análisis y posteriormente diferentes grupos trabajaron en optimizarlo y aplicarlo al tamizaje metabólico en neonatos ^8^^,^^9^ incluyendo la adición de succinilacetona (SUAC), como un marcador específico para la tirosinemia tipo I (Tyr I) ^10^^,^^11^, la MS/MS actualmente reemplaza las técnicas de tamizaje tradicionales que analizan biomarcadores individuales para cada enfermedad. En la actualidad, la MS/MS se utiliza para tamizaje neonatal en países desarrollados como Estados Unidos, Canadá, Alemania y España ^12^^-^^15^, y en países de América Latina como México, Brasil y Costa Rica ^16^^-^^18^. En Colombia no existe suficiente conciencia sobre este tema, lo que conduce a una falta de claridad en cuanto a las políticas de salud pública para su manejo ^19^. Actualmente, sólo el hipotiroidismo congénito está incluido en la lista de enfermedades obligatorias para el tamizaje, sin embargo, se sabe que aún la prueba no se realiza en muchas de las regiones del país.

Debido a la necesidad urgente de un método altamente sensible para el diagnóstico y el tamizaje eficiente de los EIM en Colombia, este estudio se enfoca en el establecimiento de valores de concentración de aminoácidos (AAs), acilcarnitinas (ACs) y succinilacetona (SUAC) normales en una muestra colombiana de recién nacidos utilizando como tecnología la MS/MS.

## Materiales y Métodos 

### Sujetos

Un total de 891 recién nacidos sanos de Cali (n = 523, departamento del Valle del Cauca) y Quibdó (n = 368, departamento de Chocó) fueron reclutados entre agosto de 2012 y julio de 2015 e incluidos en el estudio. Las muestras de sangre fueron colectadas por punción en talón e impregnadas en papel de filtro (filtro Whatman nº903, GE Healthcare, Westborough, EE.UU.), luego se dejaron secar durante 24 horas a temperatura ambiente y posteriormente fueron almacenadas a 4º C hasta su uso, siguiendo las guías del Instituto de Normas Clínicas y de Laboratorio (CLSI) ^20^. Las muestras sobrantes fueron almacenadas y congeladas a -20º C con desecante. 

Este ensayo se realizó de acuerdo con las Directrices para Buenas Prácticas Clínicas de la ICH E-6 y el protocolo fue aprobado por los comités de ética del Centro Internacional de Vacunas- CIV (CECIV de Cali, acto 005), Clínica Versalles (Cali) y el Hospital San Francisco de Asís (Quibdó). Se obtuvo el consentimiento informado por escrito (CI) de la madre de cada recién nacido al momento de la inscripción al estudio.

### Criterios de Inclusión

Todos los niños incluidos en el estudio cumplieron con todos los criterios de inclusión para asegurar que no sufrían ningún trastorno o enfermedad. Los recién nacidos sanos de ambos sexos debían cumplir criterios como tener pesos entre 2,500 y 4,000 g, edades gestacionales entre 37 a 42 semanas, puntuación APGAR mayor de 7 a los 10 min y edad entre 2 y 18 días de nacidos.

### Materiales 

Estándares de AAs y AC marcados isotópicamente fueron adquiridos en Cambridge Isotope Laboratories, Inc .; la sal de sulfato de hidrazina y acetonitrilo de grado HPLC en Sigma-Aldrich, SUAC de HT Brink, del Hospital Universitario de Ámsterdam, el ácido fórmico y HCl 3 N n-butanol de Fluka. El papel de filtro 903 Protein Saver usado para muestras de muestras de sangre se compró a Whatman. Los controles basales, de baja, media y alta concentración en sangre fueron proporcionados por el Programa de Aseguramiento de Calidad de tamizaje neonatal (NSQAP) del Centro de Control y Prevención de Enfermedades (CDC) (Lote 1421-1424; 1462-1464). Las muestras de sangre secas se analizaron usando cromatografía líquida de alta eficacia (HPLC) (Shimadzu Scientific Instruments, Columbia, MD) acoplada a un espectrómetro de masas en Tandem 3200 QTRAP (AB Sciex).

### Preparación de la muestra

Discos de sangre seca (DSS) se perforaron a un diámetro de 3.2 mm, y cada uno se dispuso en un pozo de una placa de poliestireno de 96, a los cuales se adicionaron 120 µL de solución de trabajo preparada diariamente que contenía: acetonitrilo:agua (80:20 v/v), 0.08% de ácido fórmico, 3.6 mol/L de hidrato de hidracina y los estándar internos de AAs, ACs y SUAC. La placa fue sellada con microfilm autoadhesivo (Fisherbrand No 08-408-240) y agitada a 600 rpm a 45° C durante 45 min. 

Los extractos fueron transferidos a una nueva placa de poliestireno de 96 pozos y fueron secados bajo atmosfera de N_2_ a temperatura ambiente por aproximadamente 25 min a 40° C. Después se adicionaron 50 µL de metanol y los pozos de nuevo se secaron durante 10 min. Las muestras fueron reconstituidas en 60 µL de n-butanol 3N HCl e incubadas a 65° C ±5° C por 20 min. La mezcla resultante se secó nuevamente durante 20 min, y los residuos fueron finalmente reconstituidos en 150 µL de fase móvil (acetonitrilo:agua 50:50 con 0.025% de ácido fórmico), la placa fue cubierta con papel aluminio, agitada por 10 min a temperatura ambiente, y centrifugada durante 4 min a 500 rpm antes de ser puesta en la bandeja del automuestreador para el análisis por MS/MS.

### Análisis por MS/MS

Un espectrómetro de masas en tándem con analizadores en triple cuadrupolo lineal, operado en modo ión-positivo fue usado para el análisis de AAs, ACs y SUAC. Las muestras se analizaron usando como fase móvil acetonitrilo:agua 50:50 con 0.025% de ácido fórmico en modo isocrático, a un flujo de 70 µL/min en una corrida de 3 min por muestra usando una bomba binaria HPLC (Shimadzu Scientific Instruments, Columbia, MD). 

El análisis fue realizado usando tres experimentos diferentes por corrida: 1. Perdida neutra de m/z 102 (rango de scan m/z: 130/280) para la detección de AAs, 2. Precursor iónico de m/z 85 (rango del scan 200-550) para ACs, y 3. Modo de reacción múltiple (MRM) para SUAC (m/z 211 → 137) y los AA con baja sensibilidad como la Arg (m/z 231 → 70), Gly (m/z 132 → 76), Leu (m/z 188 → 86), Met (m/z 206 → 104), Ser (m/z 162 → 60), His (m/z 156 → 110), OH-Pro (m/z 188 → 68), Thr (m/z 176 → 74) Ornitine (m/z 189 → 70) y Citruline (m/z 232 → 113). El software Analyst 1.5.2 (BioSciex) fue usado para la adquisición de los datos, y los programas Chemoview y Neoscreen fueron usados para el análisis de los datos. La cuantificación de los analitos de interés se realizó calculando la abundancia de iones de cada compuesto puro en relación con los estándares internos marcados isotópicamente (IS).

### Linealidad y límite de detección

La linealidad del método para AAs, ACs y SUAC se estimó por duplicado mediante el análisis de los controles en DSS del CDC, a cuatro concentraciones diferentes entre 3.7 y 746.7 µmol/L para AAs, 0.1 y 52.8 µmol/L para ACs y 0.3 y 11.1 µmol/L para SUAC y a un intervalo de confianza (IC) del 95%. El límite de detección (LOD) se evaluó utilizando una linealización de los tres niveles de concentración inferior para cada analito, teniendo en cuenta el ruido de base como indicador de la sensibilidad del instrumento.

### Precisión y exactitud

Tanto la repetibilidad como la precisión intermedia del ensayo para AAs, ACs y SUAC se estimaron a dos niveles de concentración (baja y media) de los controles en DSS del CDC. La repetibilidad se determinó por duplicado en cinco repeticiones, mientras que la precisión intermedia fue determinada por duplicado durante tres días y dos analistas diferentes. La precisión fue denotada como el coeficiente de variación (CV <20%) y valores de Cochran (<0.555) para la repetibilidad y reproducibilidad respectivamente.

La precisión del ensayo se determinó utilizando los controles en DSS del CDC a cuatro niveles de concentración (Lot: 1321-24 para AA y Lot: 1361-64 para AC) en tres series de corridas. La precisión se denominó como el error relativo (%RE), y debía estar dentro de ±20% RE para ser aceptado ^21^.

### Análisis estadístico

Los datos se procesaron con R-project de Bell Laboratories (antes AT&T, ahora Lucent Technologies). Los resultados se expresan como coeficientes de correlación (R^2)^ para la linealidad, valor crítico de la distribución F (análisis ANOVA) para el análisis de los valores de varianza, CV y Cochran para la precisión y porcentaje de recuperación para la exactitud. Se utilizó la prueba de Shapiro-Wilk para comprobar la normalidad; sin embargo, no hubo pruebas suficientes para decir que los datos se distribuyeran normalmente. Las comparaciones entre grupos por sexo y lugar del estudio se determinaron mediante la prueba U Mann Whitney y las correlaciones entre la edad y concentración de los analitos se determinaron usando la prueba de Spearman. Un valor de *p* ≤0.05 se consideró significativo.

## Resultados

### Validación del método 

La linealidad del método se determinó utilizando cuatro niveles de concentraciones. Los coeficientes de regresión lineal (R²) para AAs, ACs y SUAC fueron >0.96 para todos los analitos (Tabla 1). Los rangos de linealidad se encontraban dentro de los rangos establecidos por el CDC. El análisis de varianza para la regresión mediante la prueba ANOVA mostró valores críticos de la distribución F muy inferiores al 5% para todos los analitos (Tabla 1).

**Tabla 1:** Validación del método

Analito	^*^LOD (µmol/L)	Rango lineal (µmol/L)	Linealidad	ANOVA	Precisión		Exactitud
			**R^2^	*Valores críticos* †*F*	¶CV	Cochran	§% ER
			≥0.95	<5%	≤ 20%	0.555	80 - 120%
Phe	0.10	71.8 - 325.4	0.998	3.399E-05	8.17	0.43	83.24
XLeu	1.43	116.0 - 445.7	0.966	2.681E-03	10.39	0.245	91.06
Met	4.97	11.4 - 214.2	0.980	1.236E-03	14.65	0.309	96.59
Tyr	0.33	49.7 - 489.4	0.993	2.567E-04	6.18	0.356	98.63
Val	2.27	121.4 - 428.6	0.991	3.389E-04	6.7	0.534	92.47
Cit	0.35	26.3 - 232.7	0.999	3.050E-07	4.78	0.455	109.94
Ala	1.50	292.8 - 625.5	0.978	1.374E-03	6.17	0.25	105.2
Arg	0.89	10 - 266.1	0.995	1.358E-04	5.9	0.484	84.34
SUAC	0.12	1.4 - 8.98	0.995	1.318E-04	6.78	0.374	104.79
Carnitine libre (C0)	0.04	25.9 - 79	0.971	2.104E-03	9.28	0.277	92.58
Acetil (C2)	0.08	12.9 - 39.4	0.939	6.500E-03	10.95	0.22	83.78
Propionil (C3)	0.14	1.3 - 12.4	0.999	8.953E-06	8.63	0.384	114.48
Butiril (C4)	0.09	0.2 - 4.4	0.992	3.003E-04	13.19	0.297	94.55
Isovaleril (C5)	0.01	0.1 - 2.4	0.999	7.922E-06	5.07	0.544	105.48
Glutaril (C5DC)	0.15	0.5 - 1.8	0.997	6.015E-05	13.64	0.336	88.96
3-OH-valeril (C5OH)	0.01	0.5 - 3.4	0.964	2.975E-03	5.05	0.304	79.77
Hexanoil (C6)	0.01	0.0 - 2.2	0.999	2.258E-06	8.82	0.554	113.21
Octanoil (C8)	0.06	0.0 - 2.3	0.983	9.102E-04	2.8	0.353	116.59
Decanoil (C10)	0.03	0.0 - 2.5	0.981	1.165E-03	7.37	0.332	113.11
Dodecanoil (C12)	0.01	0.0 - 2.1	0.991	3.672E-04	12.37	0.366	100.13
Myristoil (C14)	0.01	0.1 - 2.6	0.997	6.014E-05	12.66	0.467	107.1
Palmitoil (C16)	3.47*	0.9 - 9.2	0.958	3.752E-03	8.14	0.33	113.3
Hydroxy-Palmitoil (C16OH)	2.83*	0.0 - 0.9	0.995	1.664E-04	8.17	0.43	175.4
Estearoil (C18)	4.58*	0.6 - 5.7	0.996	9.952E-05	10.39	0.245	91.06
OH-Estearoil (C18OH)	3.75*	0.0 - 1.4	0.987	6.520E-04	14.65	0.309	96.59

* LOD: Límite de detección

* *R2: Coeficiente de correlación

†F: Valor crítico de distribución F

¶CV: Coeficiente de variación

§%ER: Error relativo

La exactitud del método se determinó mediante el análisis de muestras que contenían cantidades conocidas de cada analito en controles en DSS del CDC. Los datos de precisión obtenidos estuvieron en el rango de 83.2 a 109.9% para AAs, de 80.0 a 116.6% para ACs y 104.8% para SUAC (Tabla 1). Los LODs obtenidos para AA, AC y SUAC también se resumen en la Tabla 1, mostrando la detección más baja para fenilalanina (Phe) (0.1 µmol/L) y para la 3-hidroxipalmitoil-carnitina (C16OH) (2.83 nmol/L).

### Concentraciones de AAs, ACs y SUAC en la población de estudio

Se incluyeron en este estudio un total de 891 neonatos sanos de entre 1 y 18 días de nacidos, 463 (52%) hombres y 428 (48%) mujeres. De ellos, 313 recién nacidos tenían entre 1 y 2 días, 257 entre 3 y 8 días y 321 entre 9 y 18 días. Las muestras en DSS se usaron para determinar los 25 analitos validados, y que forman parte de los controles de calidad entregados por el CDC, adicionalmente, se analizaron otros 32 analitos para completar un total de 57, de los cuales 17 eran AAs, 37 ACs y SUAC, que permiten identificar 50 patologías diferentes. Los marcadores específicos se seleccionaron de acuerdo con la información obtenida a partir de la página de mejoramiento de calidad de laboratorios de tamizaje neonatal de Region 4 ^22^ y que fueron transferidos desde el Hospital Clínico Universitario, Santiago de Compostela, España al Centro Internacional de Vacunas, Cali. 

En general, los AAs (n= 8, Tabla 2) tenían concentraciones medias que oscilaban entre 15 y 247 µmol/L. De ellos, xLeu (media 247 µmol/L), Ala (192 µmol/L) y Val (128 µmol/L) fueron los AAs más abundantes, mientras que Citrulina (15 µmol/L) y Arg (20 µmol/L) presentaron las concentraciones más bajas. Por otra parte, las ACs con cadenas más cortas fueron las más concentradas, mientras que las de cadenas más largas fueron las menos abundantes. Se observaron las concentraciones más altas para la carnitina libre (32 µmol/L) y acetil carnitina (23 µmol/L), mientras que la hidroxiestearoil carnitina (C18-OH) (0.07 µmol/L) fue la menos abundante. La Palmitoyl carnitina (C16) fue la de mayor concentración entre las ACs de cadenas largas (Tabla 2).

**Tabla 2:** Valores de concentración de referencia para concentración de aminoácidos (AAs), acilcarnitinas (ACs)

Analito	Media (µmol/L)	DS	^*^P_1_	^**^P_99_
Phe	44.27	11.78	23.92	78.94
XLeu	246.72	121.16	72.61	536.72
Met	35.50	19.71	11.00	101.91
Tyr	96.27	34.72	37.03	201.25
Val	128.42	32.10	59.18	204.00
Cit	14.95	4.56	7.81	27.52
Ala	192.01	53.86	91.43	348.82
Arg	19.94	11.48	4.40	52.07
C0	32.39	13.71	13.63	78.74
C2	22.69	8.62	9.02	54.31
C3	1.24	0.73	0.23	4.05
C4	0.22	0.15	0.07	0.64
C5	0.24	0.09	0.09	0.59
C5DC	0.12	0.07	0.03	0.36
C5OH	0.22	0.09	0.10	0.55
C6	0.16	0.09	0.04	0.51
C8	0.16	0.08	0.05	0.43
C10	0.17	0.09	0.05	0.45
C12	0.27	0.16	0.08	0.81
C14	0.32	0.15	0.10	0.84
C16	2.48	1.19	0.72	6.17
C16OH	0.1	0.06	0,02	0,27
C18	0.83	0.36	0.30	2.00
C18OH	0.07	0.05	0.02	0.26

DS: Desviación estándar

* Percentile 1: límite de aceptación inferior

* *Percentile 99: límite de aceptación superior

### Distribución por edad y sexo

Se compararon las concentraciones normales de AAs y ACs con respecto a factores como la edad y el sexo. Los datos no mostraron una distribución normal cuando fueron evaluados usando la prueba de normalidad de Shapiro-Wilk, por lo que se utilizaron pruebas no paramétricas para verificar las diferencias entre concentraciones de AAs y ACs con respecto a los diferentes factores.

La concentración de analitos tuvo una correlación significativa, con un rho que osciló entre 0.02 y 0.52 para las relaciones positivas y -0.06 y -0.39 para las relaciones negativas (Tabla 3). Para los AAs, sólo la fenilalanina y la metionina tuvieron una correlación negativa (Fig. 1A), mientras que para los ACs, en todos los casos excepto el Isovaleril (C5, rho=0.02) la correlación entre la concentración y la edad fue negativa (rho <0) (Fig. 1B, Tabla 3), indicando que la concentración de estos analitos disminuye ligeramente con la edad, mientras que aumenta con la edad para el isovaleril y la mayoría de los AAs.

**Tabla 3:** Análisis estadístico por edad (Correlación de Spearman) y sexo (Prueba de Mann Whitney U).

Analito	Correlación de Spearman		Niña		Niño		Prueba de Mann Whitney U
	rho	valor *p*	Mediana (µmol/L)	IQR*	Mediana (µmol/L)	IQR1	valor *p*
Phe	-0.38	9.30E-21	42.34	36.14-49.44	42.21	36.09-49.43	0.842
XLeu	0.49	5.05E-35	216.36	149.29-335.02	213.10	149.91-347.67	0.677
Met	-0.21	8.99E-07	30.42	21.25-43.53	28.83	21.5-40.04	0.633
Tyr	0.46	6.78E-31	93.84	72.67-115.06	92.17	71.08-115.74	0.467
Val	0.50	2.10E-37	127.77	106.77-150.94	128.84	105.28-147.00	0.535
Cit	0.05	2.03E-01	14.06	12.02-17.00	14.30	12.12-16.89	0.955
Ala	0.30	1.69E-13	190.78	155.77-229.23	183.80	155-217.67	0.139
Arg	0.52	2.24E-40	16.94	9.73-26.00	18.83	10.83-29.60	0.018
C0	-0.29	1.45E-12	28.34	22.24-36.87	30.92	24.46-38.66	0.004
C2	-0.28	1.90E-11	21.03	17.30-26.59	20.95	17.18-25.61	0.484
C3	-0.12	4.96E-03	1.04	0.78-1.44	1.10	0.82-1.48	0.133
C4	-0.10	1.81E-02	0.20	0.15-0.27	0.20	0.15-0.26	0.798
C5	0.02	6.19E-01	0.23	0.17-0.28	0.23	0.18-0.28	0.945
C5DC	-0.32	4.95E-14	0.10	0.06-0.14	0.10	0.07-0.15	0.107
C5OH	-0.10	1.29E-02	0.20	0.16-0.26	0.21	0.16-0.26	0.252
C6	-0.31	1.26E-13	0.14	0.10-0.18	0.14	0.10-0.20	0.573
C8	-0.31	2.32E-13	0.14	0.10-0.19	0.14	0.11-0.20	0.251
C10	-0.35	1.10E-17	0.15	0.10-0.20	0.15	0.11-0.21	0.086
C12	-0.38	1.57E-20	0.22	0.15-0.32	0.23	0.16-0.34	0.557
C14	-0.31	1.99E-14	0.28	0.21-0.37	0.31	0.22-0.39	0.024
C16	-0.39	2.23E-22	2.22	1.61-3.06	2.25	1.70-3.12	0.310
C16OH	-0.36	3.95E-17	0.08	0.05-0.13	0.08	0.06-0.12	0.560
C18	-0.36	3.97E-19	0.75	0.56-0.99	0.75	0.59-1.00	0.727
C18OH	-0.28	2.16E-10	0.06	0.04-0.09	0.06	0.04-0.09	0.938

*IQR es el rango entre el percentil 25 y 75

**Figura 1:** Diagramas de dispersión para algunos AAs. A. Aminoácidos (fenilalanina, metionina y leucina) y B. carnitina de cadena corta (carnitina libre e isovalerilcarnitina) y carnitina de cadena larga (palmitoilcarnitina) por edad. Se resaltan la media, mediana, los percentiles 1º y 99º y la regresión lineal. Cada analito tiene una escala diferente, todos ellos medidos en µmol/L.

Cuando se hicieron comparaciones por sexo, la arginina fue el único aminoácido que mostró diferencias significativas (*p*= 0.018, mayor concentración en los hombres), mientras que en el caso de los AC, la carnitina libre y la miristoil carnitina también presentaron diferencias significativas con concentraciones más altas en los hombres, con valores *p* de 0.004 y 0.024, respectivamente (Fig. 2, Tabla 3).

**Figura 2:** Diagramas de caja y bigotes por sexo para la arginina, la carnitina libre y la miristoilcarnitina. Las cajas corresponden al percentil 25 y 75 y la línea horizontal es la concentración media del analito.

## Discusión 

Aquí reportamos una metodología para evaluar simultáneamente 57 analitos relacionados con AAs, ACs y SUACs, asociados con más de 40 EIMs utilizando la MS/MS a partir de DSS recolectada de recién nacidos de Colombia. Esta metodología permitió determinar los valores normales de concentración de estos analitos en una población étnicamente diversa de 891 recién nacidos. Basado en la tasa nacional de natalidad (663,908 nacimientos/año) ^23^, este tamaño de muestra representa una población estadísticamente significativa para el establecimiento de valores normales de los metabolitos analizados y cumple con las guías del Instituto de Normas Clínicas y de Laboratorio (CLSI) ^24^.

Se observó que el método mostró una correlación lineal en una amplia gama de concentraciones, así como límites de detección bajos cuando se evaluaron muestras de sangre enriquecidas con los analitos de estudio preparados en DSS (controles de calidad del CDC), y posteriormente mezclados con estándares internos marcados isotópicamente. En general, no se observaron problemas mayores en la toma y manejo de las muestras, en menos del 10% de las muestras se presentaron problemas de calidad de los DSS, secado insuficiente o retrasos en tiempo de entrega. Los parámetros de validación establecidos mostraron una gran precisión y exactitud, que se evidencia en los CVs y los porcentajes de recuperación que se encuentran dentro de los valores aceptados internacionalmente para métodos bioanalíticos ^21^. 

Las variables utilizadas en la regresión para cada analito fueron estadísticamente significativas con valores críticos de la distribución F <5% (prueba ANOVA), demostrando por lo tanto que el modelo implementado fue adecuado. Las concentraciones calculadas a partir de las muestras de recién nacidos mostraron los límites más altos para AAs y más bajos para ACs y SUAC, lo que se correlaciona con una mayor ingesta de proteínas durante la alimentación del lactante.

Cuando se comparan los percentiles 1 y 99 con los reportados por Región 4 genetics para estos metabolitos, se observan valores en rangos similares para la mayoría de ellos usando una población mucho más pequeña. Algunas carnitinas, entre ellas la Hidroxi-octadecanoilcarnitina (C18), mostraron percentiles más altos (99) en comparación con los reportados por otros laboratorios, lo que podría representar un problema debidos al posible aumento de falsos negativos al superponerse entre el corte superior de lactantes sanos y el corte inferior en lactantes enfermos.

Aunque las concentraciones medias de ACs estuvieron en un rango bajo de 0.07 -32.4 µmol/L, éstas estuvieron siempre por encima de su LOD, lo cual es importante para la identificación de pacientes con algunos trastornos metabólicos ^25^. Los perfiles de acilcarnitina en DSS de recién nacidos se caracterizaron, en la mayoría de los casos, por una disminución en la concentración a medida que el niño crecía, acorde con datos reportados en estudios previos ^26^.

En el caso de la isovaleril carnitina, que mostró un ligero aumento de la concentración con la edad, podría atribuirse su variabilidad a factores externos como el uso de antibióticos o la hemólisis relacionada con la ictericia. La edad debe considerarse como un factor importante en la determinación de los valores de concentración de referencia, teniendo en cuenta las dificultades relacionadas con el diagnóstico en los lactantes de más edad debido a la disminución de las concentraciones de ACs. Los valores de corte más bajos para estos analitos en niños mayores podrían conducir a una dificultad en el diagnóstico de trastornos metabólicos que muestren deficiencia de carnitina. 

No se encontraron diferencias significativas entre niños y niñas para la mayoría de los metabolitos, excepto en el caso de la arginina, la carnitina libre y la miristoilcarnitina, que mostraron una mayor concentración para los neonatos hombres, estos resultados se correlacionan con estudios previamente reportados para las carnitinas ^27^^,^^28^.

Se observaron diferencias significativas entre las concentraciones en DSS de los analitos cuando se compararon los resultados entre Cali y Quibdó (datos no mostrados). Sin embargo, también hubo diferencias en la edad media de los recién nacido captados en las dos poblaciones, mientras que los recién nacidos de Cali tenían una media de 9 días (rango: 4-18 días), los recién nacidos de Quibdó tenía una edad media de 2 días (rango: 1-7 días) de nacidos. Se sabe que la edad, las terapias farmacológicas, la nutrición parenteral, las transfusiones y el tipo de alimentación pueden influir potencialmente en los resultados. Estas diferencias se tuvieron en cuenta al establecer los valores límite. 

El uso de MS/MS para la detección de EIM en recién nacidos ofrece algunas ventajas como la sensibilidad analítica, selectividad y precisión, con la posibilidad de medir varios analitos en un solo análisis con una tasa de interferencias relativamente baja ^29^. La mayoría de las limitaciones del método que se presenta aquí, están relacionadas con la calidad de los DSS, la toma de muestras de sangre, los efectos cromatográficos y volumétricos, la estabilidad del analito y los efectos de hematocrito ^30^, que en la mayoría de los casos, puede resolverse mediante la estricta adherencia a las guías clínicas y de laboratorio ^20^.

Se ha sugerido que la integridad de las muestras de sangre seca en papel de filtro puede verse comprometida en un corto periodo de tiempo por la humedad y la temperatura a la que son expuestas durante el transporte de las muestras, se puede presentar una degradación significativa de los AA y AC en las muestras de sangre a alta temperatura (45° C) y humedad (>70%), particularmente en el primer día de almacenamiento ^31^. Por lo tanto, se deben controlar las condiciones de transporte y almacenamiento de las muestras. Por otra parte, la mayoría de metabolitos utilizados para la evaluación EIM en el recién nacido dependen del hematocrito y de la posición de corte en el disco ^32^^,^^33^. Aunque los niveles de hematocrito en neonatos son significativamente variables (45-60%), en la mayoría de los casos las concentraciones de metabolitos exceden inequívocamente sus respectivos niveles de corte y el hematocrito y/o la localización del disco no afectan significativamente el diagnóstico. Sin embargo, es importante aclarar que dentro de la metodología de nuestro estudio, no se midió el hematocrito antes de las pruebas metabólicas.

En conclusión, este es el primer reporte de análisis de los metabolitos característicos para el diagnóstico de EIM por MS/MS en Colombia, tal como se realiza en los países desarrollados. Actualmente, las pruebas bioquímicas y las herramientas analíticas que se utilizan en Colombia no tienen suficiente sensibilidad y especificidad en comparación con la MS/MS. Además, las técnicas como la cromatografía de gases y los métodos enzimáticos y colorimétricos utilizan protocolos complejos con un tratamiento prolongado de las muestras y se restringen a un número limitado de analitos asociados con unas pocas enfermedades. La aplicación de tecnología avanzada, como el análisis automatizado de MS/MS con ionización en electrospray, permite la detección de más de 40 EIM con una reducción significativa del tiempo de análisis (2-3 min).

Los datos reportados aquí representan una contribución significativa para establecer los niveles normales de concentración de AAs, ACs y SUACs para ser usados como referencia para la implementación de un programa de tamizaje metabólico de recién nacidos en Colombia basado en la tecnología MS/MS, que permita definir la prevalencia nacional de estas EIM.
